# A high-quality sponge gourd (*Luffa cylindrica*) genome

**DOI:** 10.1038/s41438-020-00350-9

**Published:** 2020-08-01

**Authors:** Haibin Wu, Gangjun Zhao, Hao Gong, Junxing Li, Caixia Luo, Xiaoli He, Shaobo Luo, Xiaoming Zheng, Xiaoxi Liu, Jinju Guo, Junqiu Chen, Jianning Luo

**Affiliations:** Guangdong Key Laboratory for New Technology Research of Vegetables, Vegetable Research Institute, Guangdong Academy of Agricultural Sciences, Guangzhou, Guangdong 510640 China

**Keywords:** Plant evolution, Structural variation

## Abstract

Sponge gourd (*Luffa cylindrica*) is an important cultivated vegetable and medicinal plant in the family Cucurbitaceae. In this study, a draft genome sequence of the sponge gourd inbred line P93075 was analyzed. Using Illumina, PacBio, and 10× Genomics sequencing techniques as well as new assembly techniques such as FALCON and chromatin interaction mapping (Hi-C), a chromosome-scale genome of approximately 656.19 Mb, with an N50 scaffold length of 48.76 Mb, was generated. From this assembly, 25,508 protein-coding gene loci were identified, and 63.81% of the whole-genome consisted of transposable elements, which are major contributors to the expansion of the sponge gourd genome. According to a phylogenetic analysis of conserved genes, the sponge gourd lineage diverged from the bitter gourd lineage approximately 41.6 million years ago. Additionally, many genes that respond to biotic and abiotic stresses were found to be lineage specific or expanded in the sponge gourd genome, as demonstrated by the presence of 462 *NBS-LRR* genes, a much greater number than are found in the genomes of other cucurbit species; these results are consistent with the high stress resistance of sponge gourd. Collectively, our study provides insights into genome evolution and serves as a valuable reference for the genetic improvement of sponge gourd.

## Introduction

Sponge gourd [*Luffa cylindrica* (L.) Roem (*L. cylindrica*), syn. *L. aegyptiaca* Mill] is a dicotyledonous vine species belonging to the family Cucurbitaceae that originates in tropical Asia. It is an important vegetable and medicinal plant in tropical and subtropical regions globally^1,2^. Several recent studies have revealed that sponge gourd is a good source of carbohydrates, vitamin C, various minerals (i.e., Mg, Ca, Na, K, Fe, Cu, Zn, and Mn)^3,4^, tannin, oxalate, phytin phosphorus, and phytic acid^5^, indicating its potential as a source of vegetable protein in human diets^4^. Sponge gourd has also been widely used in medicine. Alcalase or tryptic protein hydrolysates in its seeds are an effective treatment for diabetes and hypertension^6^. Additionally, the leaves, seeds, and fruits of sponge gourd have been used for the treatment of various diseases, including inflammatory diseases, diarrhea, and viral infections^7,8^, and the triterpenoids isolated from sponge gourd (sapogenins 1 and 2) exhibit immunomodulatory activity^9^. These findings explain the high medicinal value of sponge gourd, making it a focus of recent scientific research.

The lack of reference genome sequences is a major obstacle to basic and applied biology research in *Luffa*^10,11^. In the present study, we generated a high-quality assembly of the sponge gourd genome. The whole-genome sequence of sponge gourd was generated using the Illumina, PacBio, Hi-C, and 10× Genomics GemCode sequencing platforms, followed by de novo assembly. The final genome was 656.19 Mb (the N50 values of the contig and scaffold lengths were 8.80 and 48.76 Mb, respectively) and contained 25,508 protein-coding genes, and 63.81% of the genome was occupied by repetitive elements. The sponge gourd genome was compared with the genomes of other species in Cucurbitaceae such as *Benincasa hispida*^12^*, Citrullus lanatus*^13^*, Cucumis melo*^14^*, Cucumis sativus*^15^*, Cucurbita moschata*^16^*, Cucurbita pepo*^17^*, Lagenaria siceraria*^18^, and *Momordica charantia*^19^ to analyze its evolution. The results presented in this study will be valuable for biosynthesis studies seeking to affirm the medicinal value of sponge gourd as well as plant breeding research for the genetic improvement of sponge gourd.

## Results

### Genome sequencing, assembly, and quality evaluation

**Fig. 1:**
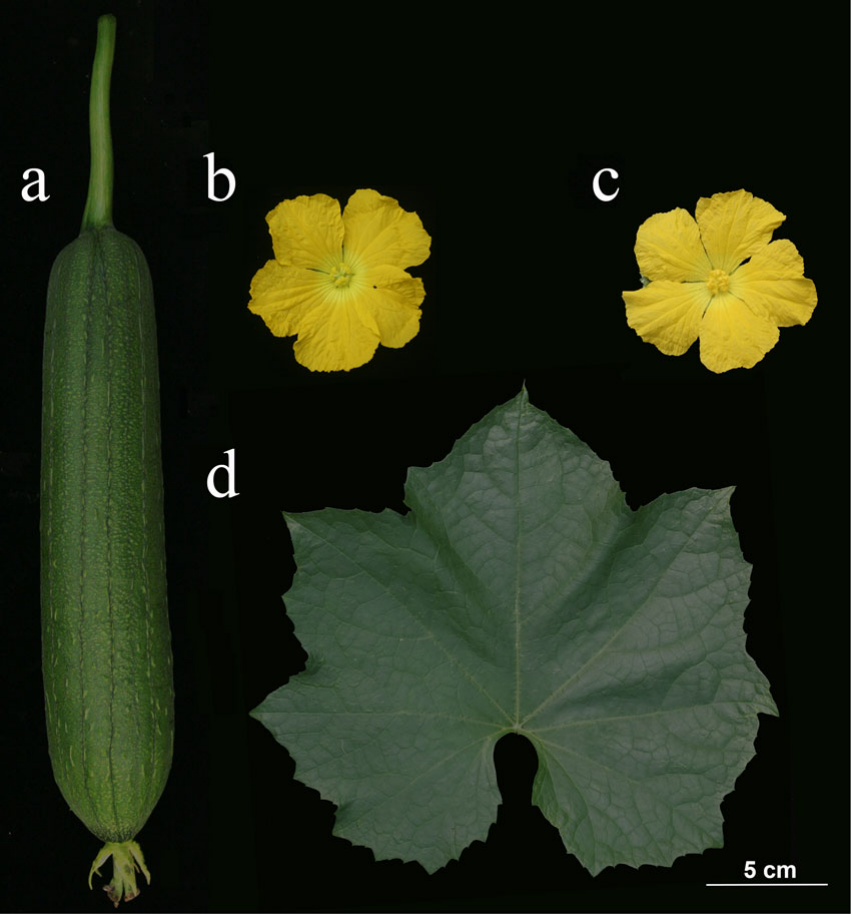
Morphological characteristics of the sponge gourd inbred line P93075. **a** Fruit. **b** Female flower. **c** Male flower. **d** Leaf

**Fig. 2:**
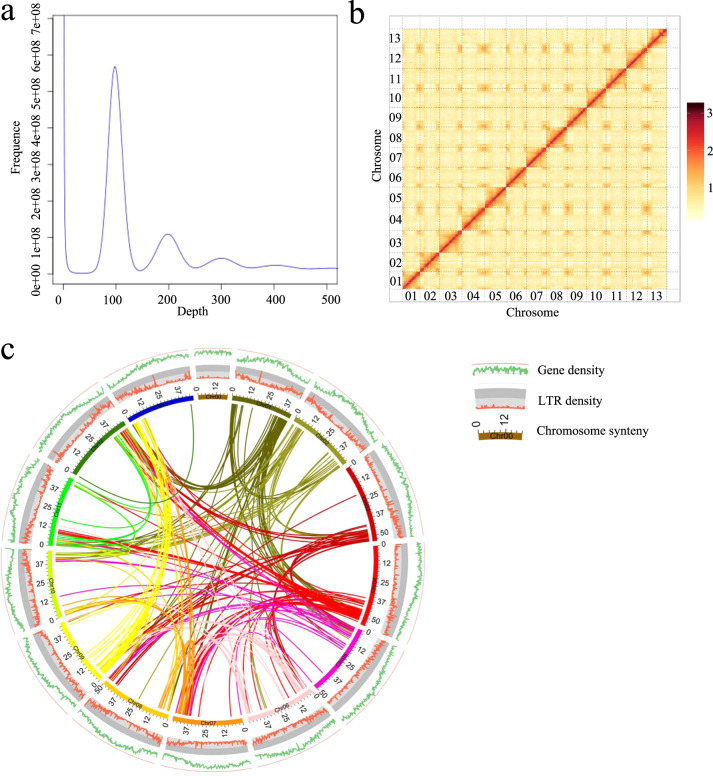
Sponge gourd genome assembly. **a** The results of *k*-mer analysis used to estimate the size of the sponge gourd genome. **b** Hi-C contact map data analysis. **c** Circular diagram showing the genetic collinearity among sponge gourd chromosomes. The concentric circles (from inside to outside) are as follows: gene density, LTR density, and collinear genome blocks, which are connected by curved lines of the same color for each element. All data are rendered based on a 200-kb window size

To assess the genome size of the sponge gourd inbred line P93075 (Fig. 1, Supplementary Fig. 1), 144.34 Gb of paired-end reads (with an insert size of 350 bp) were selected to generate 17-mer frequency data for *k*-mer analysis. The *k*-mer depth distribution was plotted against the *k*-mer frequency, with the highest peak occurring at a depth of 97 (Fig. 2a). Based on the total number of *k*-mers (71,593,662,168 bp), the sponge gourd genome size was calculated to be ~720.33 Mb, with genome heterozygosity of 0.06% (Supplementary Table S1). Then, the genome was assembled using PacBio single-molecule real-time (SMRT) sequencing (92.53 Gb of raw data), 10× Genomics sequencing (87.90 Gb of raw data), and Hi-C chromosome-scale scaffolding (87.36 Gb of raw data). The assembly consisted of 328 scaffolds, with an N50 scaffold length of 18.61 Mb (total length of 656.80 Mb) and an N50 contig length of 9.44 Mb (total length of 655.84 Mb). Subsequently, the Hi-C sequencing data were aligned to the assembled scaffolds using BWA-mem^20^; the complete genome was 656.19 Mb, and the N50 values of the contig and scaffold lengths were 8.80 and 48.76 Mb, respectively (Table 1). Finally, the scaffolds were anchored onto 13 chromosomes (Fig. 2b), and the average length of the chromosomes was 48.9 Mb, among which the shortest was 42.2 Mb (Chr01), and the longest was 55.6 Mb (Chr04) (Supplementary Table S2).

**Table 1:** Summary of the final assembly of the sponge gourd genome

	Illumina + 10× Genomics+ PacBio+ Hi-C			
Sample ID	Length		Number	
	Contig(bp)	Scaffold(bp)	Contig	Scaffold
Total	655,835,779	656,189,986	480	332
Max	22,699,861	55,641,800	–	–
Number≥2 kb	–	–	462	315
N50	8,800,239	48,760,765	25	7
N60	6,922,768	48,278,130	33	8
N70	4,570,118	47,313,622	45	10
N80	3,331,685	46,820,663	61	11
N90	1,493,572	46,425,688	91	12

A map connecting homologous regions of the genome is shown in Fig. 2c. Overall, 99.51% of the raw reads could be mapped to the assembly, which indicates that our assembly includes almost all of the information contained in the raw reads. The analysis of core eukaryotic genes revealed homologs of 91.53% of the conserved genes in the assembly (Supplementary Table S3). The genome was also assessed using the BUSCO^21^ gene set, which includes 2121 single-copy orthologous genes, and the results indicated that 95.5% of the conserved genes were found in the sponge gourd genome (Supplementary Table S4). Approximately 99.51% of the reads could be mapped to the assembly, which covered 99.74% of the assembled sequence (Supplementary Table S5). In summary, all these results support the high quality of the assembled sponge gourd genome.

### Genome annotation

**Fig. 3:**
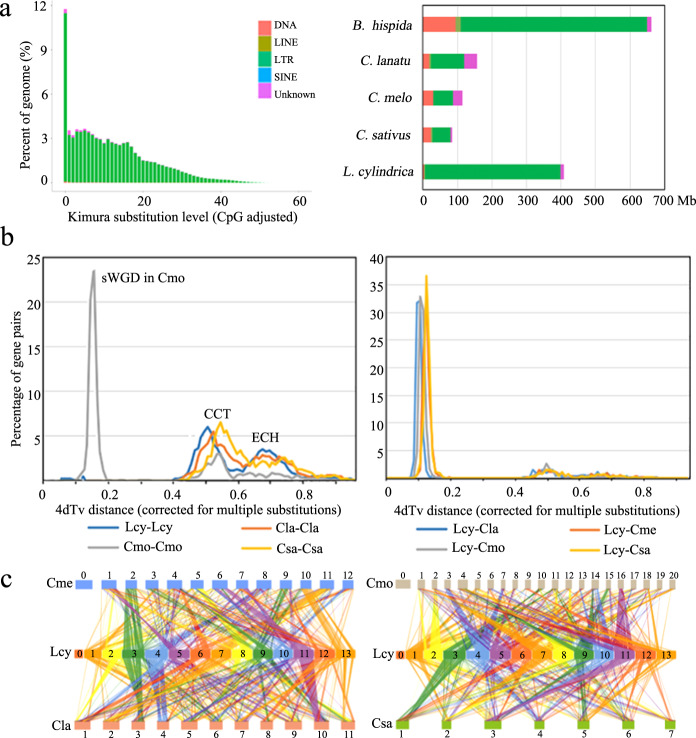
Whole-genome duplication and genetic collinearity analysis. **a** Distribution of transposable elements in the sponge gourd genome. **b** Whole-genome duplication (WGD) events detected in the genomes of *Luffa cylindrica* (Lcy), *Cucumis sativus* (Csa)*, C. melo* (Cme)*, Citrullus lanatus* (Cla), and *Cucurbita moschata* (Cmo), with the distribution of transversion substitutions at fourfold degenerate sites (4dTv) shown on the left and the divergence time between Lcy and other species shown on the right. **c** Schematic representation of synteny between *L. cylindrica* and *Cucumis sativus, C. melo, Citrullus lanatus*, and *Cucurbita moschata*. Each line connects a pair of orthologous genes between genomes

Tandem repeats were detected in the genome using Tandem Repeats Finder (TRF)^22^. Ultimately, repetitive sequences accounted for 63.81% of the sponge gourd genome (Table 2). Additionally, 62.62% of the genome was occupied by transposable elements (TEs), and long terminal repeats (LTRs) constituted the most abundant category of TEs, occupying 60.69% of the genome (Fig. 3a, Table 2, Supplementary Table S6). We also predicted 781 miRNA genes, 1592 transfer RNA genes, 4682 small nuclear RNA genes, and 302 ribosomal RNA genes in the sponge gourd genome (Supplementary Table S7). To predict protein-coding genes, we used homology-based prediction, de novo prediction, and transcriptome-based prediction based on transcriptomics analysis results derived from samples of the roots, leaves, flowers, fruits, and stems of sponge gourd. In total, 27,154 genes were predicted from the sponge gourd genome (Supplementary Table S8). Through a combination of ab initio prediction, homology searches, and RNA sequence-aided prediction, 25,508 protein-coding genes were predicted, 93.90% of which were functionally annotated (Supplementary Table S9). The average transcript and CDS lengths were 4184.44 bp and 1160.18 bp, respectively. The average exon and intron lengths were 241.63 bp and 795.55 bp, respectively, with 4.8 exons per gene on average. By mapping the RNA reads onto the annotated genome, we found that the majority of the RNA reads (>86%) from the five sponge gourd tissues could be mapped to annotated exon regions, which were associated with the transcription of 19,739 genes.

**Table 2:** Summary of repeat content in the sponge gourd genome

Type	Length (bp)	Percent (%)
Trf	47,476,933	7.27
Repeatmasker	406,062,229	61.82
(Gypsy)	(173,056,987)	(26.35)
(Copia)	(160,654,933)	(24.46)
Proteinmask	88,261,982	13.44
Total^a^	419,095,893	63.81

^a^“Total” is the nonredundant result obtained via the above methods after removing the overlapping regions between the different approaches

### Whole-genome duplication analysis

Whole-genome duplication (WGD) events have been common throughout plant evolution, playing a crucial role in the evolution and speciation of plants. To investigate WGDs in the sponge gourd lineage, we identified syntenic regions across the sponge gourd, cucumber (*Cucumis sativus*), melon (*Cucumis melo*), watermelon (*Citrullus lanatus*), and pumpkin (*Cucurbita moschata*) genomes. Based on the transversion substitutions identified in the fourfold degenerate sites (4DTv = 0.6) of collinear gene pairs, the core eudicot-common hexaploidy (ECH) event (occurring 115–130 MYA) and ancient cucurbit-common tetraploidy (CCT) event (occurring 90–102 MYA)^23^ could be identified (Fig. 3b). Similar to the cucumber, melon, and watermelon lineages, there was no lineage-specific whole-genome duplication (sWGD) observed in the sponge gourd lineage (Fig. 3b), but this contrasted with the results in the pumpkin lineage, which underwent a sWGD event after the CCT event^23^. Further synteny analysis provided a robust sequence framework for understanding the genome evolution of sponge gourd and was used to explore the factors underlying genome expansion (Fig. 3c). Chromosomes 3, 4, 7, and 11 of the sponge gourd genome showed the greatest synteny with the chromosomes of the other four cucurbitaceous plants, demonstrating that these chromosomes exhibit lower rearrangement rates. Sponge gourd, cucumber, melon, watermelon, and pumpkin exhibited highly conserved synteny, although extensive chromosomal rearrangement was found to have occurred in these species.

### Comparative genomics

To investigate the evolution of the sponge gourd genome, we compared it with the genomes of 13 other sequenced species, including *Citrullus lanatus, Cucumis melo, C*. *sativus, Cucurbita moschata, C*. *pepo, Lagenaria siceraria*, *Benincasa hispida*, and *Momordica charantia*, which are cucurbitaceous species, and *Arabidopsis thaliana, Vitis vinifera, Solanum lycopersicum*, and *Glycine max*, as outgroups. Compared with *M*. *charantia, Cucurbita moschata*, and *C*. *pepo*, 2695 genes (1221 families) were unique to sponge gourd (Supplementary Table S10). The GO classification of these unique genes showed that they were enriched in the DNA replication, metabolism of DNA, ATP, and carbohydrate derivatives, proton transport, biotic stimulus, and defense response categories (Supplementary Table S11).

**Fig. 4:**
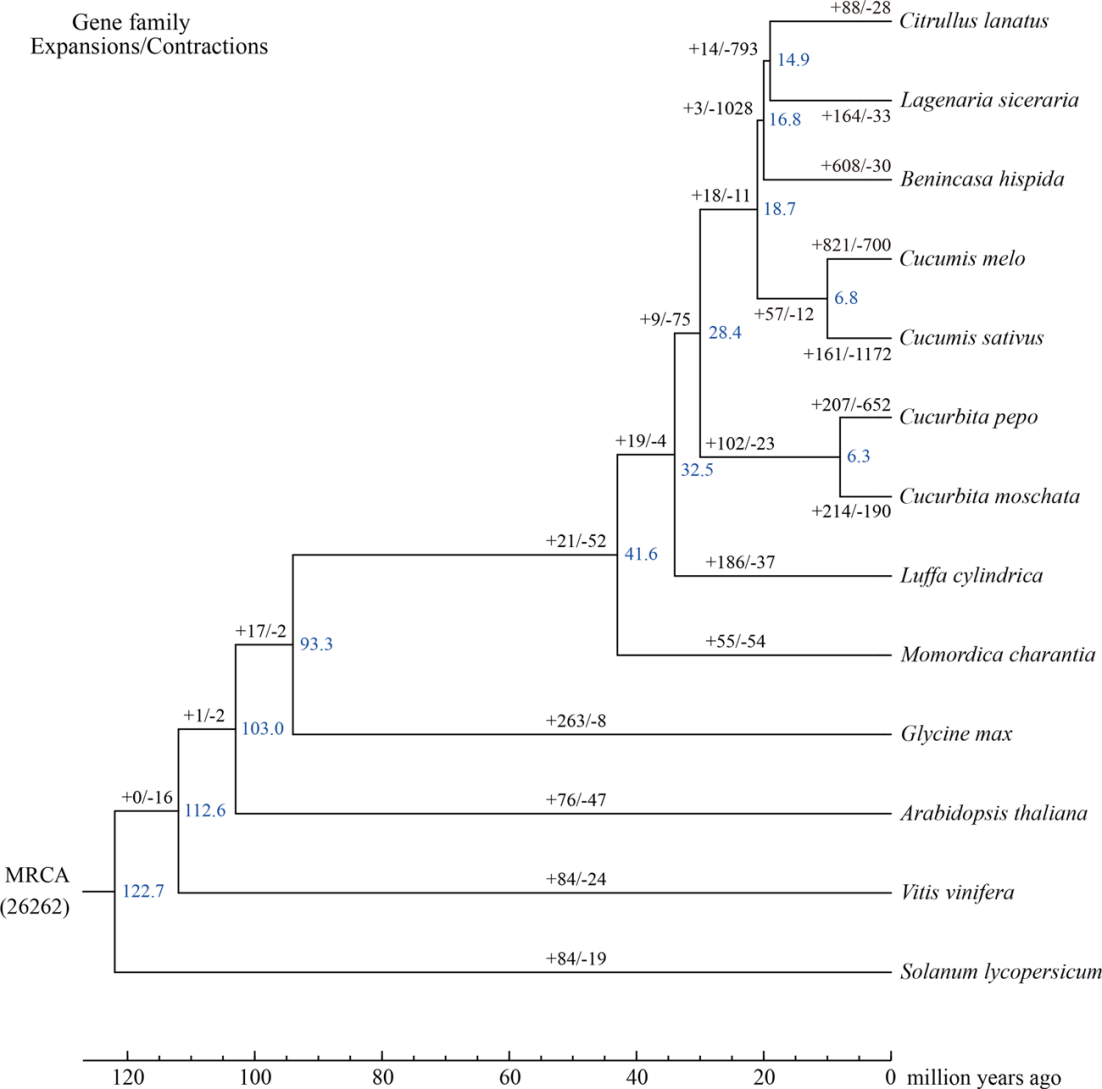
Gene family expansions and contractions and the estimated divergence time in sponge gourd and 12 other species. Blue numerical value beside each node shows the estimated divergence time (MYA, million years ago). The numbers on the left and right of the slash indicate expanded and contracted gene families, respectively

Further phylogenetic analysis allowed the divergence times between sponge gourd genes and their homologs in the other plants to be estimated, indicating that the sponge gourd lineage (Fig. 4) diverged from the bitter gourd lineage (*M. charantia*) approximately 41.6 million years ago, with subsequent divergence from other cucurbitaceous plants occurring approximately 32.5 million years ago. Expansions and contractions of orthologous gene families were also determined, revealing 186 expanded gene families and 37 contracted gene families in the sponge gourd lineage (Fig. 4). The GO classification of expanded gene families (Supplementary Table S12) revealed enrichment for genes involved in nucleic acid metabolic and defense response processes.

Notably, many of the lineage-specific genes and expanded genes found in sponge gourd were enriched in stress response-related GO terms (Supplementary Table S12) and KEGG pathways (Supplementary Table S13), including the response to stress (GO:0006950), defense response (GO:0006952), and biosynthesis of antibiotics (map01130). Toll and interleukin-1 receptor (TIR) is an N-terminal component of the nucleotide binding site (NBS) disease resistance protein family^24,25^, which includes the TIR-NBS-LRR (TNL) and CC-NBS-LRR (CNL) subfamilies and is associated with ADP binding (GO:0043531) and the defense response (GO:0006952). The expanded gene families in the sponge gourd genome were significantly enriched for GO:0043531 and GO:0006952. Moreover, among the 25,508 annotated sponge gourd reference genes, 462 genes (Supplementary Table S14) encoding enzymes and NBS-LRR domains were identified. Additionally, various copies of NBS-LRR were tandemly duplicated in sponge gourd, including FRAGSCAFF37.822(*Lcy05g001660*)/825(*Lcy05g001640*)/828(*Lcy05g001620*)/829(*Lcy05g001610*)/835(*Lcy05g001570*)/837(*Lcy05g001550*)/839(*Lcy05g001530*). Considering that no sWGD event occurred after the CCT event in the sponge gourd lineage, we inferred that the expansion of *NBR-LRR* genes has been a major cause of genome evolution through lineage-specific tandem duplications.

The fibers of sponge gourd fruit are widely used around the world. Many studies have shown that the main components of loofah fibers are cellulose, hemicellulose, and lignin^26^. To understand the regulation of the formation of sponge gourd fibers, we analyzed genes associated with the cellulose, hemicellulose, and lignin synthesis pathways based on genomic annotation. Notably, chitinase-like (CTL), caffeoyl CoA 3-*O*-methyltransferase (CCoAMT), cinnamoyl CoA reductase (CCR), caffeoyl shikimate esterase (CSE), ferulic acid/coniferaldehyde 5-hydroxylase (F5H), and laccase (LAC) genes in sponge gourd were significantly expanded compared with those in other cucurbitaceous crops, except for pumpkin, which underwent a sWGD event after the inferred CCT event (Supplementary Table S15).

## Discussion

In this study, we produced a high-quality genome sequence for sponge gourd. By combining 10× Genomics, PacBio, and Hi-C sequencing data, we were able to assemble a genome sequence of 656.19 Mb, which covers ~91.2% of the sponge gourd genome, and these sequences were anchored to 13 protochromosomes. The N50 values of contig and scaffold lengths were 8.80 and 48.76 Mb, respectively, and the genome included 95.5% of the conserved BUSCO core gene set. Thus, this assembly is an almost complete representation of sponge gourd genome and provides a valuable reference for the study of important agronomic characteristics of sponge gourd and related species.

### The evolution and genome characteristics of sponge gourd

The sponge gourd genome (656.19 Mb) is substantially larger than that of most other sequenced cucurbitaceous species (269–469 Mb)^13–19^ but smaller than that of wax gourd (913 Mb)^12^. Sponge gourd shows much more obvious TE expansion (62.62%) than other cucurbitaceous species such as watermelon (45.2%)^13^, melon (14.7%)^14^, cucumber (24.4%)^15^, and bitter gourd (15.3%)^19^ but similar TE expansion to wax gourd (66.18%)^12^. Accordingly, this expansion may have played a crucial role in the increase in the genome size of sponge gourd.

Synteny analysis between the sponge gourd genome and those of the other four cucurbitaceous species also showed differences in the rearrangement frequency among these chromosomes. There was high collinearity between Chr 3 of the sponge gourd genome and Chr 2 of melon, Chr 3 of watermelon, Chr 10 and Chr 11 of pumpkin (consistent with the sWGD observed in pumpkin), and Chr 1 of cucumber (Fig. 3c). Interestingly, the genes located in high-collinearity regions were highly enriched in the carbohydrate metabolic process (GO:0005975, such as *Lcy03g002220, Lcy03g015120*, and *Lcy03g017280*) and ATPase activity categories (GO:0016887, such as *Lcy03g000500*, *Lcy03g007020*, and *Lcy03g013190*), showing that these basic developmental regulatory genes have been relatively well conserved in these five cucurbitaceous plants during their evolution.

Further WGD event analysis revealed ECH (115–130 MYA) and CCT (90–102 MYA) events (Fig. 3b), and pumpkin was also determined to have undergone a sWGD event^16^ that did not occur in the sponge gourd genome, verifying the accuracy of our findings. Taken together, these results show that the expansion of repetitive sequences (especially LTRs) was a crucial contributor to the genome expansion of sponge gourd. This was consistent with the GO classification results for the expanded genes (Supplementary Table S12), which showed high enrichment for genes associated with DNA replication and metabolism, such as DNA polymerase^27^ activity-related genes (*Lcy04g003450, Lcy04g003830* and *Lcy04g005130*). Notably, LTR expansion has also played a crucial role in the genome evolution of wax gourd, with insert expansion occurring approximately 7–11 MYA^12^, after the divergence (based on the phylogenetic analysis results) of sponge gourd from other cucurbitaceous plants (32.5 MYA) as well as the divergence of wax gourd from *Cucumis* (16.8 MYA) (Fig. 4). Therefore, it can reasonably be speculated that adaptive evolution in the wax gourd lineage resulted from selection on phenotypic variation associated with TE insertions occurring approximately 10 MYA. Further group evolution analysis with different cultivars collected from representative regions could be performed to identify evolutionary bottleneck events and the patterns of evolution among the different cucurbitaceous species.

### Disease defense-related family expansion

Throughout evolution, plants experience the expansion and contraction of gene families, which are changes that underlie phenotypic evolution. Sponge gourd is much more resistant to stress, including biotic stress and abiotic stress, than other cucurbitaceous species and has been widely used as a rootstock to improve crop yields, overcome soil-borne diseases, and enhance flooding tolerance^28,29^. Many expanded and lineage-specific gene families in sponge gourd were enriched for involvement in responses to biotic stress and/or abiotic stress, such as the defense response, the response to biotic stimuli, plant–pathogen interactions, and plant hormone signal transduction, which play important roles in defense against potential biotic and/or abiotic stresses. Furthermore, there were many more *NBS-LRR* genes identified in sponge gourd (462) than in the genomes of *Benincasa hispida*^12^, *Citrullus lanatus*^13^, *Cucumis melo*^14^, *Cucumis sativus*^15^, *Cucurbita maxima*^16^, and *Cucurbita moschata*^16^ (Supplementary Table S14). All of these results are consistent with the high biotic and abiotic stress resistance of sponge gourd.

### Preliminary analysis of genes associated with nutrition in sponge gourd

Sponge gourd is an important cultivated vegetable that is rich in nutrients essential for human health, including vitamin C, arginine, and phosphorus^3,30^. The Gene Ontology analysis results for specific gene families in sponge gourd were enriched for transferase activity related to the transfer of phosphorus-containing groups (GO:0016772) (Supplementary Tables S11 and 12), while the corresponding KEGG analysis showed significant enrichment of genes involved in arginine biosynthesis (Supplementary Table S13). Phosphorus plays crucial roles in plant growth and development by regulating plant hormone transport^31,32^. Furthermore, arginine is important for the development of plants (especially root systems)^33^ and is the precursor for the synthesis of endogenous hormone polyamines, thus improving tolerance to high salinity and other stress conditions^34,35^; these roles are consistent with the enrichment of specific and expanded gene families in genes involved in the defense response (Supplementary Tables S11 and S12). These results indicate the genomic changes that underlie the value of sponge gourd as a traditional edible vegetable.

### Initial genome-level study on the medicinal value of sponge gourd

In addition, the GO analysis of expanded gene families showed the enrichment of genes involved in the cellular aromatic compound metabolic process category (GO:0006725) (Supplementary Table S12), which is consistent with the richness of sponge gourd in flavonoids (high content in leaves), triterpene alcohols, *trans*-ocimene, α/β-pinene, β-myrcene (high content in flowers), and many other chemical monomers and medicinally active compounds^36^. Flavonoids, including formononetin, genistein, and isoliquiritigenin, which are effective in cancer prevention or therapy^37,38^, have been reported to be the main bioactive components^38,39^. Although genes involved in flavonoid biosynthesis have been identified in *Arabidopsis*^40,41^ and leguminous plants^42^, an overall understanding of the genes involved in flavonoid biosynthetic pathways in sponge gourd is lacking. Weighted gene coexpression network analysis (WGCNA)^43^ has contributed to the identification genes involved in the flavonoid synthesis pathway of sponge gourd, which will be assessed through analyses of RNA-Seq results in our next research project. Further biosynthesis studies are crucial for affirming the medicinal value of sponge gourd and promoting its commercial viability.

### Expansion of genes involved in the synthesis of cellulose, hemicellulose, and lignin

The synthesis of cellulose, hemicellulose, and lignin in plants is complex. CTLs are likely to play a key role in establishing interactions between cellulose microfibrils and hemicelluloses, thus affecting cellulose biosynthesis^44^. Specifically, l-phenylalanine is converted to lignin by deamination (PAL), hydroxylation (C3H, C4H, HCT), *O*-methylation (COMT, CCoAMT), CoA activation (4CL), and reduction (CCR, CAD)^45^. The genes involved in these processes were expanded in sponge gourd (Supplementary Table S15), which may be responsible for the formation of fibers in sponge gourd fruits.

## Conclusion

Using Illumina, PacBio, 10× Genomics, and chromatin interaction mapping (Hi-C) data, a chromosome-scale genome of ~656.19 Mb in length was generated, with 25,508 protein-coding genes. This high-quality chromosome-scale assembly and functional annotation of the sponge gourd genome will facilitate evolutionary studies of the family Cucurbitaceae and the identification of candidate genes related to natural medicinal substances. Our research provides novel information that is relevant for comparative genome studies involving sponge gourd. Finally, the sponge gourd genome provides a solid foundation for future studies, not only in sponge gourd but also in other cucurbitaceous species.

## Materials and methods

### Plant materials

P93075, an advanced inbred line of sponge gourd, was used for genome sequencing. Young leaves were collected and immediately frozen in liquid nitrogen. For RNA extraction, fresh plant tissues, including roots, leaves, flowers, fruits, and stems, were collected, and external contaminants were removed by washing the samples with ultrapure water three times.

### DNA extraction and Illumina library preparation

Sponge gourd genomic DNA was extracted from young leaf tissue using the DNAsecure Plant Kit (TIANGEN, Beijing, China). Sequencing libraries with 350-bp inserts were constructed using a library construction kit (Illumina, San Diego, CA, USA) and then sequenced using the Illumina HiSeq X Ten platform.

### PacBio library construction and sequencing

A 20-kb-insert size DNA library was constructed. Then, PacBio libraries were sequenced on the PacBio Sequel platform (Pacific Biosciences, Menlo Park, CA, USA).

### 10× Genomics library construction and sequencing

DNA sample preparation was conducted using a GemCode Instrument from 10× Genomics (Pleasanton, CA, USA). A DNA sample of 1 ng was used for the GEM reaction procedure based on PCR. The library was finally sequenced using the Illumina HiSeq X Ten platform.

### Hi-C library construction and sequencing

DNA from young leaves was fixed to generate the Hi-C library. The leaf cells were lysed, and the *Hind* III endonuclease was used to digest the fixed chromatin. Then, the sheared 350-bp fragments were ligated to adaptors^46^ and labeled with biotin. After PCR enrichment, the libraries were sequenced using the Illumina HiSeq X Ten platform.

### Estimation of genome size using *k*-mer analysis

To estimate genome characteristics, *k*-mer frequency analysis was used^47^. The genome size of P93075 sponge gourd was calculated based on *k*-mer (*k* = 17) statistics.

### Genome assembly

The de novo assembly of the long reads from the PacBio SMRT Sequencer was performed by using FALCON (https://github.com/PacificBiosciences/FALCON/) and FALCON-Unzip^48^. To obtain sufficient corrected reads, the longest segment with at least 60× depth (longest coverage of subreads) was selected for sequence error correction. Then, the error-corrected reads were assembled into genomic contigs with FALCON. After the initial assembly, FALCON-Unzip was used to produce primary contigs (p-contigs), which were then polished using Quiver^49^. Next, we used BWA-MEM to align the 10× Genomics data to the assembly^20^. The construction of scaffolds was performed using fragScaff with the barcoded sequencing reads. Finally, error correction was performed using Pilon^26^ based on the Illumina sequences. Subsequently, the Hi-C sequencing data were aligned to the scaffolds using BWA-mem^20^, and the scaffolds were clustered onto chromosomes using LACHESIS (http://shendurelab.github.io/LACHESIS/).

### Genome annotation

RepeatModeler (http://www.repeatmasker.org/RepeatModeler.html)^50^ and LTR_FINDER (http://tlife.fudan.edu.cn/ltr_finder/) were used for de novo repeat family identification^51^, and RepeatScout (http://www.repeatmasker.org/) was used to build the de novo repeat library. For the homology-based approach, RepeatMasker (http://www.repeatmasker.org, version 4.0.5) and RepeatProteinMask (http://www.repeatmasker.org/) were used against the Repbase TE library and TE protein database^52^. TRF^22^ was used to detect tandem repeats in the sponge gourd genome.

For transcriptome-based prediction, RNA-seq data were mapped to the sponge gourd genome using TopHat (version 2.0.8) and Cufflinks (version 2.1.1)^53,54^. In addition, Trinity was used to assemble the RNA-seq data, and the resulting assembly was used to generate several pseudounigenes, which were mapped to the assembly. Finally, PASA (http://pasapipeline.github.io/)^55^ was used to predict the gene models.

BLASTP (E-value = 1e-05) analysis against the SwissProt and NR databases^56^ was used for functional annotation. InterProScan (V4.8) and HMMER (V3.1) analyses were performed against the InterPro and Pfam databases, respectively^57–60^. The tRNA genes were identified by using tRNAscan-SE software^61^. rRNA fragments were predicted by alignment to rRNA sequences based on BLASTn analysis (E-value of 1e-10). INFERNAL^62^ and the Rfam database (release 9.1)^63^ were used to predict miRNAs and snRNAs.

### Gene collinearity analysis

Matched genes with E-values < 1e^_^5 were considered as candidate homologs. Next, ColinearScan^64^ was performed to identify syntenic blocks. Whole-genome duplication (WGD) analysis was performed using MCScanX software (http://chibba.pgml.uga.edu/mcscan2) with the default parameters. Then, 4DTv distances were calculated for each gene pair in each syntenic block.

### Comparative genome analysis

Comparative analysis was performed to identify orthologous gene families among the 13 plant species as described in the main text, including sponge gourd. For all-against-all protein BLAST searches, we first filtered out the proteins that were fewer than 50 amino acids in length and retained the longest protein among alternative splice variants. Then, we used BLASTP (E-value < le-7) for searches of the filtered proteins, and we clustered them into orthologous groups using OrthoMCL with the inflation parameter set at 1.5^65^. One protein per species in a cluster was clustered into the single-copy orthologs, which were used for MUSCLE alignment, and a phylogenetic tree was generated using the maximum likelihood method^66^. PAML MCMCtree (http://abacus.gene.ucl.ac.uk/software/paml.html) was used to infer the divergence time of each species. CAFÉ (https://sourceforge.net/projects/cafehahnlab) was used to analyze gene family expansion/contraction.

## Supplementary information

Supplementary tables

Supplementary table S13

Supplemental figure1

Supplementary table S11

Supplementary table S12

## Data Availability

The raw genome and transcriptome sequencing data are available from the NCBI under the project ID PRJNA596077.
