# Supplementary material for: A high-quality sponge gourd (Luffa cylindrica) genome
Source: Hortic Res. 2020 Aug 1;7:128. doi: 10.1038/s41438-020-00350-9 (PMC7395165; doi:10.1038/s41438-020-00350-9)
Supplement: Supplementary file 1 — Supplementary tables [file 41438_2020_350_MOESM1_ESM.docx]

| Supplemental Table S1. Survey statistic results of sponge gourd. | | | | | | | | | |
| --- | --- | --- | --- | --- | --- | --- | --- | --- | --- |
| Species | Total base (Gb) | K-mer | | K-mer number | K-mer depth | | Genome size (Mb) | Heterozygous ratio (%) | Repeat ratio (%) |
| sponge gourd | 144.34 | 17 | | 71,593,662,168 | | 97 | 720.33 | 0.06 | 67.23 |
| Supplemental Table S2. Length of chromosomes | | | | | | |  |  |  |
| Chromosome ID | | | Length (bp) | |  |  |  |  |  |
| Chr01 | | | 42171330 | |  |  |  |  |  |
| Chr02 | | | 46425688 | |  |  |  |  |  |
| Chr03 | | | 54102248 | |  |  |  |  |  |
| Chr04 | | | 55641800 | |  |  |  |  |  |
| Chr05 | | | 50544496 | |  |  |  |  |  |
| Chr06 | | | 48760765 | |  |  |  |  |  |
| Chr07 | | | 48278130 | |  |  |  |  |  |
| Chr08 | | | 50080546 | |  |  |  |  |  |
| Chr09 | | | 47385353 | |  |  |  |  |  |
| Chr10 | | | 46820663 | |  |  |  |  |  |
| Chr11 | | | 48955572 | |  |  |  |  |  |
| Chr12 | | | 49621835 | |  |  |  |  |  |
| Chr13 | | | 47313622 | |  |  |  |  |  |
| Average | | | 48930927 | |  |  |  |  |  |
| Total | | | 636102048 (96.9%) | |  |  |  |  |  |

| Supplemental Table S3. Assessment the gene coverage rate using CEGMA. | | | | |
| --- | --- | --- | --- | --- |
| species | complete | | complete + partial | |
|  | # Prots | %completeness | # Prots | %completeness |
| sponge gourd | 219 | 88.31 | 227 | 91.53 |

| Supplemental Table S4. Assessment the gene coverage rate using BUSCO. | |  |
| --- | --- | --- |
| Species | BUSCO notation assessment results |  |
| sponge gourd | C:95.5% [S:92.2%, D:3.3%], F:1.2%, M:3.3%, n:2121 |  |
| Size: genome size; |  | |
| BUSCO notation: C: Complete BUSCOs; S: Complete and single-Copy BUSCOs; D: Complete and duplicated BUSCOs; F: Fragmented BUSCOs; M: Missing BUSCOs; n: Total BUSCO groups searched. | | |

| Supplemental Table S5. Coverage statistics of sponge gourd genome | | |
| --- | --- | --- |
|  |  | Percentage |
| Reads | Mapping rate (%) | 99.51% |
| Genome | Coverage (%) | 99.74% |
|  | Coverage at least 4X (%) | 99.59% |
|  | Coverage at least 10X (%) | 99.49% |
|  | Coverage at least 20X (%) | 99.38% |
|  | Average sequencing depth | 184.87X |
| Average sequence depth: The average depth of each base on the genome that is covered by reads; | | |
| Coverage：The proportion of genomes that were covered by reads; | | |

| Supplemental Table S6. Summary of TE contents in sponge gourd genome. | | | |
| --- | --- | --- | --- |
| Type | Length (bp) | Percent (%) |  |
| DNA | 3,323,434 | 0.51 |  |
| LINE | 2,766,734 | 0.42 |  |
| SINE | 13,702 | 0 |  |
| LTR | 398,614,824 | 60.69 |  |
| Unknown | 11,986,162 | 1.82 |  |
| Total | 411,266,120 | 62.62 |  |

| Supplemental Table S7. The statistical results of non-coding RNA of sponge gourd genome. | | | | | |
| --- | --- | --- | --- | --- | --- |
| Type | | Copy | Average length (bp) | Total length (bp) | % of genome |
| miRNA | | 781 | 118.86 | 92,826 | 0.014133 |
| tRNA | | 1,592 | 75.38 | 120,002 | 0.018271 |
| rRNA | rRNA | 302 | 350.93 | 105,980 | 0.016136 |
|  | 18S | 103 | 762.88 | 78,577 | 0.011964 |
|  | 28S | 137 | 141.42 | 19,374 | 0.00295 |
|  | 5.8S | 37 | 156.95 | 5,807 | 0.000884 |
|  | 5S | 25 | 88.88 | 2,222 | 0.000338 |
| snRNA | snRNA | 4,682 | 109.2 | 511,260 | 0.077841 |
|  | CD-box | 4,480 | 106.47 | 476,995 | 0.072624 |
|  | HACA-box | 79 | 209.49 | 16,550 | 0.00252 |
|  | splicing | 123 | 144.02 | 17,715 | 0.002697 |

| Supplemental Table S8. Basic statistical results of gene structure prediction of sponge gourd genome. | | | | | | | |
| --- | --- | --- | --- | --- | --- | --- | --- |
| Gene set | | Number | CDS + intron length (bp) | Average CDS length (bp) | Average exon length (bp) | Average intron length (bp) | Average exons per gene |
| *De novo* | Augustus | 34,477 | 3,231.44 | 1,045.61 | 4.14 | 252.35 | 695.36 |
|  | GlimmerHMM | 50,717 | 11,024.91 | 701.42 | 3.24 | 216.7 | 4,615.08 |
|  | SNAP | 40,739 | 9,172.78 | 637.65 | 3.88 | 164.23 | 2,960.95 |
|  | Genscan | 62,058 | 4,362.65 | 685.74 | 3.33 | 206.15 | 1,580.53 |
|  | Geneid | 32,441 | 12,540.32 | 1,197.67 | 5.63 | 212.66 | 2,448.88 |
| Homolog | *Arabidopsis thaliana* | 53,663 | 1,710.62 | 779.76 | 2.4 | 324.22 | 662.54 |
|  | *Citrullus lanatus* | 58,462 | 1,522.17 | 675.74 | 2.46 | 274.82 | 580.21 |
|  | *Cucumis melo* | 41,143 | 2,640.54 | 1,099.60 | 3.25 | 338.64 | 685.74 |
|  | *Cucurbita moschata* | 22,750 | 4,571.72 | 1,420.32 | 4.38 | 323.98 | 931.28 |
|  | *Cucumis sativus* | 53,361 | 1,705.78 | 694.05 | 2.63 | 263.83 | 620.42 |
|  | *Lagenaria siceraria* | 35,407 | 3,009.11 | 920.73 | 3.34 | 275.33 | 890.89 |
|  | *Momordica charantia* | 25,162 | 4,792.35 | 1,391.30 | 4.3 | 323.79 | 1,031.57 |
| RNA-seq | Cufflinks | 77,800 | 3,657.01 | 1,018.98 | 4.85 | 210.19 | 685.59 |
|  | PASA | 51,155 | 7,140.22 | 2,292.96 | 6.76 | 338.96 | 840.85 |
| EVM | | 37,114 | 3,594.76 | 996.1 | 4.11 | 242.16 | 834.68 |
| PASA-update | | 36,865 | 3,576.28 | 1,004.21 | 4.11 | 244.17 | 826.3 |
| Final set | | 27,154 | 4,184.44 | 1,160.18 | 4.8 | 241.63 | 795.55 |

| Supplemental Table S9. The statistical results of gene function annotation of sponge gourd genome. | | | | | | | |
| --- | --- | --- | --- | --- | --- | --- | --- |
| Database | Annotated Number | Annotated Percent (%) | |  | |  |  |
| NR | 25,413 | 93.6 | |  | |  |  |
| Swiss-Prot | 19,063 | 70.2 | |  | |  |  |
| KEGG | 19,020 | 70 | |  | |  |  |
| InterPro | 21,485 | 79.1 | |  | |  |  |
| Pfam | 20,323 | 74.8 | |  | |  |  |
| GO | 14,091 | 51.9 | |  | |  |  |
| Annotated | 25,508 | 93.9 | |  | |  |  |
| Total | 27,154 | - | |  | |  |  |
|  |  |  | |  | |  |  |
| Supplemental Table S10. Genes used for gene family clustering in each species | | | | | | |  |
| Symbol | ScientificName | | Gene Number | |  |  |  |
| *Ath* | *Arabidopsis thaliana* | | 26926 | |  |  |  |
| *Cpa* | *Carica papaya* | | 26597 | |  |  |  |
| *Cla* | *Citrullus lanatus* | | 23136 | |  |  |  |
| *Cme* | *Cucumis melo* | | 19374 | |  |  |  |
| *Cmo* | *Cucurbita moschata* | | 27737 | |  |  |  |
| *Cpe* | *Cucurbita pepo* | | 28611 | |  |  |  |
| *Csa* | *Cucumis sativus* | | 23235 | |  |  |  |
| *Lcy* | *Luffa cylindrica* | | 27001 | |  |  |  |
| *Lsi* | *Lagenaria siceraria* | | 22373 | |  |  |  |
| *Mch* | *Momordica charantia* | | 18728 | |  |  |  |
| *Vvi* | *Vitis vinifera* | | 25299 | |  |  |  |
| *Sly* | *Solanum lycopersicum* | | 32775 | |  |  |  |
| *Osa* | *Oryza sativa* | | 38996 | |  |  |  |
| *Zma* | *Zea mays* | | 38986 | |  |  |  |

| Supplemental Table S14. NBS-LRR genes in different genomes | | |
| --- | --- | --- |
| Species | Number |  |
| *Benincasa hispida* | 82 |  |
| *Citrullus lanatus* | 49 |  |
| *Cucumis melo* | 84 |  |
| *Cucumis sativus* | 74 |  |
| *Cucurbita maxima* | 30 |  |
| *Cucurbita moschata* | 57 |  |
| *Luffa cylindrica* | 462 |  |

| Supplements Table S15. Genes of cellulose, hemicellulose and lignin synthesis pathway. | | | | | | |  | |  | | |  |  |  |  |
| --- | --- | --- | --- | --- | --- | --- | --- | --- | --- | --- | --- | --- | --- | --- | --- |
| Genes | Bhi | Cla | Cme | Cmo | Csa | Lcy | | Lsi | | Mch |  |  |  |  |  |
| cellulose synthase (CESA) | 31 | 36 | 36 | 48 | 31 | 28 | | 38 | | 8 |  |  |  |  |  |
| chitinase-like (CTL) | 11 | 2 | 3 | 28 | 2 | 12 | | 2 | | 2 |  |  |  |  |  |
| KORRIGAN cellulase (KOR) | 2 | 2 | 2 | 4 | 2 | 2 | | 2 | | 1 |  |  |  |  |  |
| 4-hydroxycinnamate CoA ligase (4CL) | 7 | 19 | 19 | 17 | 15 | 17 | | 6 | | 3 |  |  |  |  |  |
| coumarate 3-hydroxylase (C3H) | 1 | 1 | 1 | 1 | 1 | 1 | | 2 | | 0 |  |  |  |  |  |
| cinnamic acid 4-hydroxylase (C4H) | 6 | 4 | 6 | 5 | 3 | 4 | | 5 | | 3 |  |  |  |  |  |
| cinnamyl alcohol dehydrogenase (CAD) | 7 | 7 | 7 | 9 | 7 | 6 | | 7 | | 5 |  |  |  |  |  |
| caffeoyl CoA 3-O-methyltransferase (CCoAMT) | 3 | 2 | 2 | 5 | 2 | 7 | | 3 | | 4 |  |  |  |  |  |
| cinnamoyl CoA reductase (CCR) | 14 | 12 | 13 | 9 | 4 | 15 | | 1 | | 3 |  |  |  |  |  |
| caffeic acid/5-hydroxyconiferaldehyde 3/5-O-methyltransferase (COMT) | 7 | 1 | 3 | 3 | 4 | 5 | | 4 | | 3 |  |  |  |  |  |
| caffeoylshikimate esterase (CSE) | 10 | 4 | 6 | 8 | 9 | 10 | | 6 | | 4 |  |  |  |  |  |
| ferulic acid/coniferaldehyde 5-hydroxylase (F5H) | 2 | 3 | 3 | 2 | 2 | 3 | | 3 | | 0 |  |  |  |  |  |
| hydroxycinnamoyl CoA: shikimate hydroxycinnamoyl transferase (HCT) | 1 | 1 | 1 | 2 | 3 | 2 | | 4 | | 1 |  |  |  |  |  |
| Laccase (LAC) | 16 | 17 | 15 | 22 | 14 | 19 | | 11 | | 13 |  |  |  |  |  |
| Total | 118 | 111 | 117 | 163 | 99 | 131 | | 94 | | 50 |  |  |  |  |  |
