# Supplementary material for: A high-quality sponge gourd (Luffa cylindrica) genome
Source: Hortic Res. 2020 Aug 1;7:128. doi: 10.1038/s41438-020-00350-9 (PMC7395165; doi:10.1038/s41438-020-00350-9)
Supplement: Supplementary file 3 — Supplemental figure1 [file 41438_2020_350_MOESM3_ESM.docx]

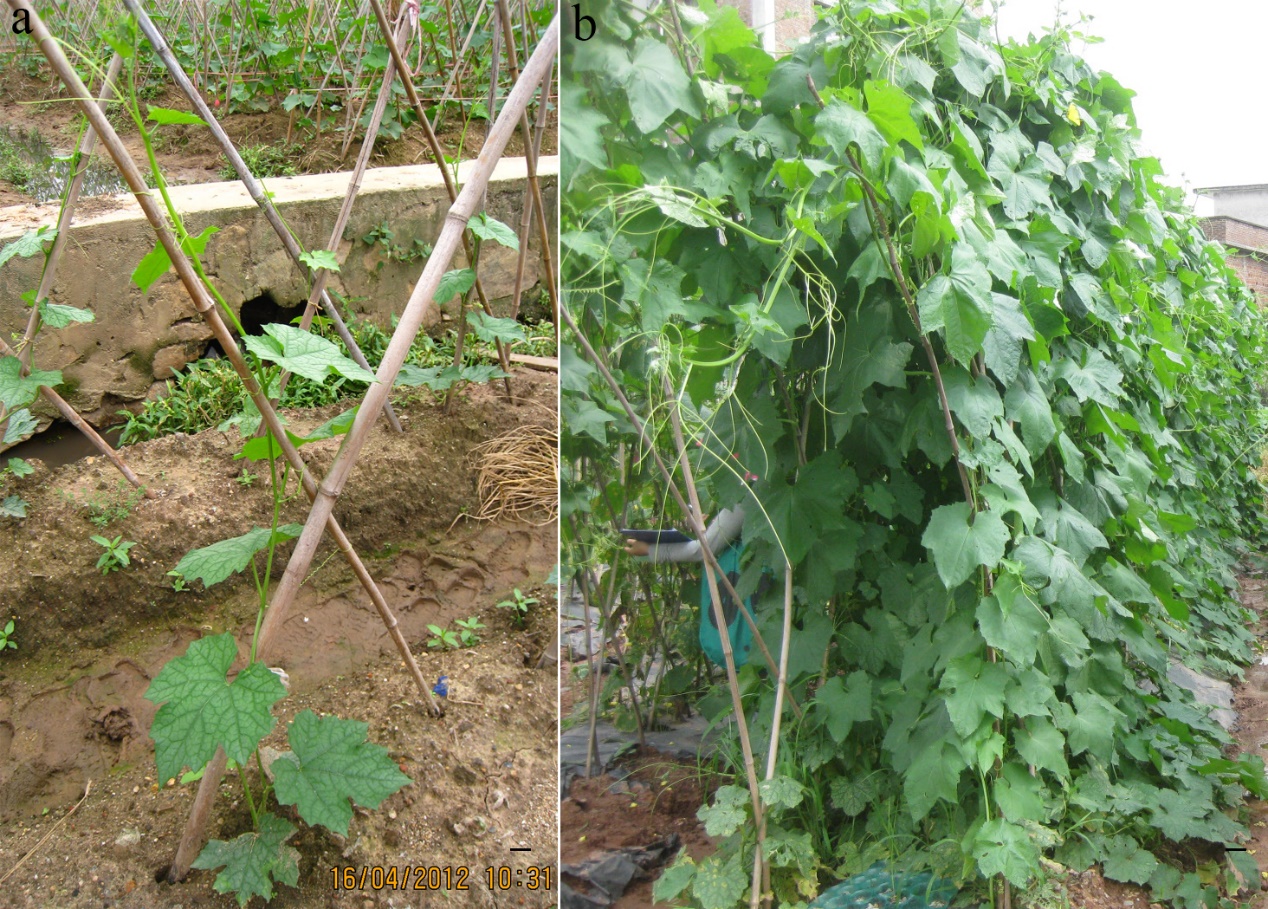


**Supplemental Fig. 1 Morphological characters of sponge gourd inbred line P93075.** **a** Seedings. Scale bar: 2 cm. **b** Adult-plant. Scale bar: 5 cm.
